# Vertebral Related Diseases in Healthcare: The Role of Pain Management and Rehabilitation

**DOI:** 10.3390/healthcare10061109

**Published:** 2022-06-14

**Authors:** Alessandro de Sire, Nicola Marotta, Antonio Ammendolia

**Affiliations:** Physical and Rehabilitative Medicine Unit, Department of Medical and Surgical Sciences, University of Catanzaro “Magna Graecia”, 88100 Catanzaro, Italy; alessandro.desire@unicz.it (A.d.S.); ammendolia@unicz.it (A.A.)

## 1. Introduction

The increase in the life expectancy of the general population implied for vertebral diseases an impacting role for the medical condition of the person, not only in the clinical context, but also from a social and economic point of view, due to the underestimation of primary prevention and complex secondary prevention rehabilitation frameworks [1]. These disabling diseases might comprise: idiopathic scoliosis, vertebral fragility fractures (VFF), and spondyloarthropathies, with unfavorable consequences for pain management, people functioning and social participation, as well as health-related quality of life (HRQoL) [2]. In this scenario, the purpose of this special issue was to frame risk factors for vertebral disorders, to provide appropriate therapeutic pathways to ensure a complete and satisfactory recovery in these patients.

## 2. Scoliosis

Adolescent Idiopathic Scoliosis (AIS) is an intricate structural disease of the spinal column that could affect a person’s physical, functional, and participatory status. Accordingly, different approaches have been introduced to recognize the curvature causes, enhancing management results [3]. The Scoliosis Research Society (SRS) defines AIS as a spine with a Cobb angle of 10° or more and accompanied by vertebral rotation [4]. Unfortunately, the etiopathogenesis of AIS remains essentially unidentified; nonetheless, current analyses reveal the likely role of genetics, estrogen, calmodulin, melatonin, vitamin D, and low bone health [5,6,7]. Similarly, AIS progression rates are the most increased among those experiencing their pubertal growth spurt, proving the presence of an asynchronous neuro-osseous maturation in AIS and other biomechanical theories. For this reason, it’s essential conducting serial clinical assessments, including the Adams bending test and extent with a scoliometer [8]. Standard radiological imaging should include the definition of the Cobb angle for diagnostic and classification purposes of the curve to provide an assessment of progression and adequate rehabilitation intervention [9,10]. Both conservative and surgical approaches are considered in the management of AIS. The brace, worn for a minimum of 18 h a day, would seem to provide good scientific evidence; furthermore, manual therapy models, such as myofascial release or spinal manipulation, might be potentially convincing in treating AIS when integrated with other conservative therapies [11]. Regardless, for subjects with angles greater than 40°, the surgical option should be considered [12]. Although spinal fusion is the traditional approach, one of the most used today, there is a recent perspective that consists of anchoring the vertebral body as a new technique to provide these adolescents with their range of motion, and therefore, a higher HRQoL [13].

## 3. Vertebral Fragility Fractures

Vertebral fractures are the most typical single osteoporotic fractures, being a critical component of the osteoporotic syndrome, a condition that affects nearly half of the elderly population [14]. Nonetheless, in contrast with hip fractures, more than two-thirds of vertebral fractures are clinically silent, and only 1 in 10 patients reach the hospital setting. On the other hand, the presence of a vertebral fracture in radiographic imaging is often omitted, rarely reported in medical records, and infrequently follows prophylactic treatment [14]. Vertebral fragility fractures, also called vertebral compression fractures, are very common due to the advancement in life expectancy [15]. In consideration of this, VFFs are combined with an increase in mortality and morbidity due to poor pain control and loss of quality of life compared to equivalents per gender and age [16]. Albeit conservative therapeutic models are widely used as first-line treatment for symptomatic vertebral fragility fractures in the elderly, a consensus on the intervention of these disorders has not been reached [17]. There isa moderate-quality evidence in favor of the use of calcitonin in the first weeks of a painful episode, but its safety profile is still uncertain, as prolonged use is related to an augmented risk of cancer [18]. The benefit of lumbar braces is promising as they present moderate but safe effects, as no serious adverse events are reported [17]. Moreover, there is inconclusive evidence to sustain vertebroplasty as some clinical trials conveyed no effect compared to control, while some studies indicated short-term improvements [19]. All the more reason, a rehabilitation program must constantly be personalized, multidimensional, and all-encompassing, looking not only at the angle of curvature or the cut-off parameters of bone health but also at the overall functioning of the patients according to a biopsychosocial model, aimed at a: (i) prevent or limit the progression curve; (ii) prevent possible fragility and disability fractures; (iii) improvement of well-being and HRQoL [20,21,22].

## 4. Spondyloarthropathies and Other Vertebral Diseases

Spondyloarthropathies (SpAs) comprise a multifactorial and heterogeneous cluster of chronic immuno-inflammatory rheumatic disorders primarily impacting the axial skeleton, but also contributing with peripheral manifestations, as well as systemic impairment (e.g., eye, heart, gut), with considerable quality of life consequences on a significant proportion of young adults causing medical, social, and economic impacts [23,24]. The prominent feature of SpAs is positive familial history and presence of HLA-B27 in most patients, which suggests genetic predisposing factors in disease pathogenesis. Ankylosing Spondylitis (AS), being the most prevalent and severe form of SpAs, is generally characterized by inflammatory low back pain accompanied by radiological findings of sacroiliitis [25]. Despite all radiological and laboratory advancements, the complicated manifestations of AS urges for multi-factorial diagnostic criteria evolved during recent years for efficient detection and follow-up of the patients [24]. According to the inflammatory base of SpAs, various anti-inflammatory medications have been used for treating the patients ranging from nonsteroidal anti-inflammatory drugs to disease-modifying anti-rheumatic drugs, including synthetic and biologic agents [26,27,28]. Nonetheless, in the biologic epoch, unmet necessities remain for uncompliant patients or biologic therapy non-responders, and despite the development of new strategies, physical therapy and rehabilitation are of crucial implication in the management of SpA patients [29].

## 5. Evidence on Pain Management and Rehabilitation for Vertebral Related Diseases

In this special issue, four manuscripts considered the primary prevention aspect of vertebral bone health and spine posture. Pharmacological approaches appear to have significant effects on fracture risk, such as selective serotonin reuptake inhibitors (SSRIs). In this context, the physician should consider the pharmacological picture to optimize their potential by reducing this risk and guaranteeing the patient an appropriate bone health [30]. Moreover, genomic pattern plays a key role in the rehabilitation scenario, as dystrophic scoliosis is mainly characterized by vertebral dysplasia and some of the other dysplastic alterations (e.g., dural ectasia, rib pencil) are associated with a higher risk of surgery [31]. Even the height of the pillow involves the cervical column alignment, correlating with the mechanical spine environment, and an adequate height of the pillow appears to provide relief by reducing cervical spine stresses, relaxing the neck and shoulder muscles, improving the quality of sleep and life [32]. A multidisciplinary approach remains essential for suitable diagnosis and posture control in people with contemporary temporomandibular disorders that might affect spine posture. The role of occlusal splints on posture is still unclear, but a converged diagnostical strategy of stabilometric and kinematic spine assessment could guarantee an adequate rehabilitation approach [33].

Four other special issue studies investigated the non-invasive treatment of spine disorders. As for conservative interventions, Schroth’s rehabilitation exercise program appears to make positive changes to idiopathic scoliosis in young adolescents [34]. In fact, several manual therapy approaches are accessible to decrease pain and disability and to recover cervical range of motion and spinal health. Muscle Energy Technique (MET) appears to show little evidence of decreasing neck pain by improving cervical range of motion in chronic disorders, even when combined with a traditional rehabilitation approach [35]. In the elderly, given that VFF are the typical form of osteoporotic fractures, a proper management is mandatory in terms of pharmacological treatment and also of appropriate prescription of spinal orthoses in both acute and chronic VFF phase [36]. Furthermore, a vertical traction approach could counterbalance the boundaries of traditional (axial) traction treatment by preserving the physiological lumbar curve, without disproportionate stress on the posterior spinal structures [37].

Three papers from the special issue investigated invasive interventions in the recovery of vertebral health. Conservative interventions are often not enough for vertebral disorders such as fractures, neoplasms or vertebral plate injuries requiring invasive options. In this scenario, vertebral body spinal tumor ablation has the conceivable to decline health care utilization by quite enhancing prompt and long-lasting developments, including a decline in opioid usage, a gain in function and HRQoL. Vertebral augmentation appears to improve in the early stages; pain, early mobilization as well as reducing the economic impact for the health system. Finally, basivertebral nerve ablation also appears to offer bearable and clinically noteworthy progress in pain and function, with some evidence reporting decreased opioid usage and disability and enhanced HRQoL [38]. Among other things, the functionality of the limbs in the quality of life and the postoperative functionality of the shoulder of cancer patients should not be underestimated after surgery with a modified muscle-sparing pectoralis major muscle flap that might guarantee a maintenance of the appropriate HRQoL [39]. Lastly, common interventions for vertebral compression fractures in osteoporotic patients are vertebroplasty and kyphoplasty, and the intervention outcomes can be associated to the weight of mixed measurement data during the procedure

Lastly, it should be underlined that machine learning analysis of preoperative clinical data on bone health, intraoperative variations and different intervention methods might provide decision-making factors, correct diagnostic-therapeutic prospects and guarantee appropriate rehabilitation approaches [22,40,41]. Indeed, a case report published on this special issue [42] reported the possibility of including the management of hematopoietic stem cells in a multidisciplinary intervention. Alongside machine learning models, another future perspective would appear to be provided by hematopoietic stem cell transplantation in vertebral compression fracture. 

**Figure 1:**
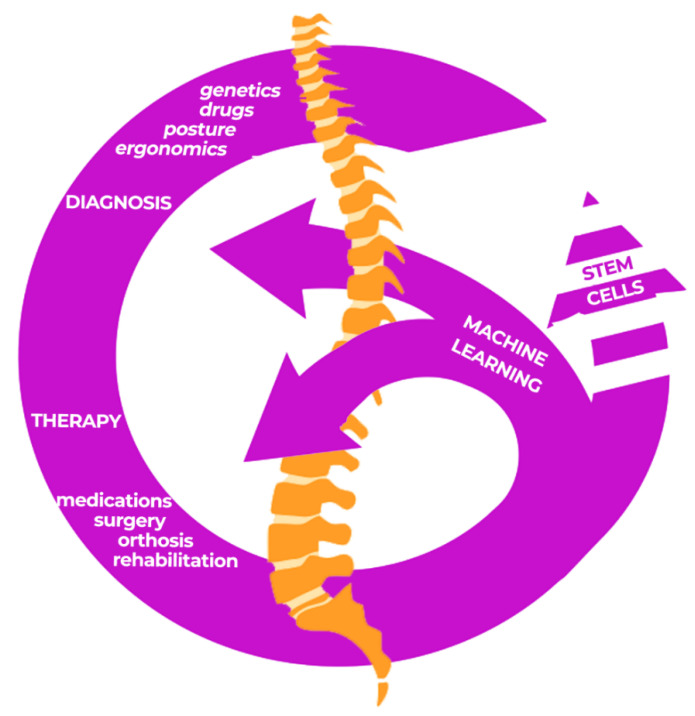
Reframing vertebral-related diseases with machine learning: a closing circle.

This could be a further future interventional option in the management of these disorders by deepening rehabilitation considerations (see Figure 1).

## 6. Conclusions

In conclusion, the papers included in the Special Issue highlighted the need for an adequate multidisciplinary approach in the therapeutic management to guarantee pain and functional recovery of patients affected by vertebral related diseases. The approaches may not necessarily be invasive and in the future new interventions, such as stem cells, could also change natural disease histories. Finally, machine learning could provide appropriate diagnostic-therapeutic models for rehabilitation for the remodeling of functions affected by vertebral disorders.

## Data Availability

Not applicable.
